# Efficacy of Invasive and Non-Invasive Brain Modulation Interventions for Addiction

**DOI:** 10.1007/s11065-018-9393-5

**Published:** 2018-12-07

**Authors:** Judy Luigjes, Rebecca Segrave, Niels de Joode, Martijn Figee, Damiaan Denys

**Affiliations:** 1 Department of Psychiatry, Academic Medical Center, University of Amsterdam, PA3.227, PO box 22660, 1100DD Amsterdam, The Netherlands; 2 Brain Imaging Center, Academic Medical Center, University of Amsterdam, Amsterdam, The Netherlands; 3 Monash Institute of Cognitive and Clinical Neurosciences, Brain and Mental Health Research Hub, Monash University, Clayton, VIC Australia; 4 Department of Psychiatry, VU University Medical Center, Amsterdam, The Netherlands; 5 Department of Psychiatry, Icahn Medical School at Mount Sinai, New York, NY USA; 6 Netherlands Institute for Neuroscience, an Institute of the Royal Netherlands Academy of Arts and Sciences, Amsterdam, The Netherlands

**Keywords:** Neuromodulation, Addiction, Deep brain stimulation, EEG neurofeedback, Transcranial magnetic stimulation, Transcranial direct current stimulation, Cognitive outcome measures

## Abstract

It is important to find new treatments for addiction due to high relapse rates despite current interventions and due to expansion of the field with non-substance related addictive behaviors. Neuromodulation may provide a new type of treatment for addiction since it can directly target abnormalities in neurocircuits. We review literature on five neuromodulation techniques investigated for efficacy in substance related and behavioral addictions: transcranial direct current stimulation (tDCS), (repetitive) transcranial magnetic stimulation (rTMS), EEG, fMRI neurofeedback and deep brain stimulation (DBS) and additionally report on effects of these interventions on addiction-related cognitive processes. While rTMS and tDCS, mostly applied at the dorsolateral prefrontal cortex, show reductions in immediate craving for various addictive substances, placebo-responses are high and long-term outcomes are understudied. The lack in well-designed EEG-neurofeedback studies despite decades of investigation impedes conclusions about its efficacy. Studies investigating fMRI neurofeedback are new and show initial promising effects on craving, but future trials are needed to investigate long-term and behavioral effects. Case studies report prolonged abstinence of opioids or alcohol with ventral striatal DBS but difficulties with patient inclusion may hinder larger, controlled trials. DBS in neuropsychiatric patients modulates brain circuits involved in reward processing, extinction and negative-reinforcement that are also relevant for addiction. To establish the potential of neuromodulation for addiction, more randomized controlled trials are needed that also investigate treatment duration required for long-term abstinence and potential synergy with other addiction interventions. Finally, future advancement may be expected from tailoring neuromodulation techniques to specific patient (neurocognitive) profiles.

## Introduction

Addiction is a burdensome public health concern that not only affects the addicted individual but also greatly impacts his or her family members and surroundings. Though several treatment options are available, many patients show a chronic course of addiction with abstinence rates between 40% and 60% 1-year post treatment (Mclellan et al. 2000). In the last few decades there is more awareness for the problem of nonsubstance related addictions such as gambling disorder (reclassified as an addictive disorder in 5th edition of the diagnostic and statistical manual of mental disorders) and excessive internet use and gaming, increasing the importance of finding new and better interventions for addiction (Banz et al. 2016).

In recent years our neurobiological knowledge of addiction has expanded. We know more about changes in brain structure, function and its neurochemistry (Fineberg et al. 2010; Koob 2015; Koob and Volkow 2009; Volkow et al. 2012). Yet, the translation of this knowledge into new interventions or improvement of existing interventions has fallen short. The non-selective manner in which effective psychotherapeutic and pharmacological interventions induce brain changes makes it difficult to apply neurobiological knowledge to improve these interventions. Neuromodulation may close the gap by targeting directly abnormalities in brain functioning. Direct intervention in the brain in order to change addiction is not new, on the contrary, neurosurgery for addiction has a long and turbulent history (Stelten et al. 2008). Yet, the advances in our understanding of brain function and the developments of new neuromodulation techniques that are non-invasive or reversible nowadays have made this intervention method a safer and more promising option.

Repetitive transcranial magnetic stimulation (rTMS) and transcranial direct current stimulation (tDCS) have been used most frequently for treatment of addiction. Other neuromodulatory options for addiction are deep brain stimulation (DBS), and neurofeedback which can be applied with Electroencephalography (EEG neurofeedback) or functional magnetic resonance imaging (fMRI neurofeedback). These techniques have been investigated as stand-alone or add on treatment and while some have been around for decades (EEG neurofeedback), others have just emerged as promising intervention for addiction (fMRI neurofeedback). In this review we will evaluate, compare and discuss the experimental application of these neuromodulation techniques for treatment of addiction.

A complicating factor in the application of neuromodulation for addiction is its heterogeneity, with large individual differences in underlying brain abnormalities (George and Koob 2010). Therefore, we choose to not only review how neuromodulation affects primary treatment outcomes for addiction such as craving or relapse, but additionally focus on how neuromodulation affects different cognitive processes involved in addiction. Cognitive processes such as reward processing, cognitive control and emotion regulation may constitute different factors underlying addiction with a closer mapping to neural abnormalities than the currently classified symptoms of addiction. One could envision that using this approach, an individually tailored type of neuromodulation that fits the cognitive profile of the patient would become possible. For instance, a highly impulsive addicted patient may receive neuromodulation focused on enhancing cognitive control in prefrontal regions whereas deeper regions like the insula or amygdala might be targeted in a patient whose addiction is driven by harm avoidance. Although the number of studies investigating the effect of neuromodulation on these cognitive processes in addiction is very limited, we will discuss them where possible.

This review aims to discuss the efficacy of neuromodulation techniques for the treatment of addiction. Although still in an early phase of development, we additionally review neuromodulatory induced cognitive changes relevant for addiction. As addictions are perpetuated by numerous maladaptive cognitive processes that can be influenced with neuromodulation techniques, this is a highly promising area for future research and treatment development. This review extends upon existing reviews addressing rTMS, tDCS (Hone-Blanchet et al. 2015; Jansen et al. 2013; Lupi et al. 2017) with additional DBS for addiction (Coles et al. 2018; Spagnolo and Goldman 2017) by (1) specifically considering only the highest quality evidence for each technique in order to evaluate the potential for clinical efficacy, (2) expanding the definition of neuromodulation to consider fMRI and EEG neurofeedback, and (3) reviewing neuromodulatory impact on addiction-related cognitive processes and discussing potential future neurocognitive targets.

## Methods

Our review focusses on studies that included adult participants who were treated for substance or behavioral addictions with neuromodulation (i.e., rTMS, tDCS, DBS, EEG & fMRI neurofeedback). With this aim we searched MEDLINE databases using PubMed for the indication terms: (addiction OR substance disorder OR craving OR gambling disorder OR binge eating disorder OR bulimia OR smoking) AND (transcranial magnetic stimulation OR repetitive transcranial magnetic stimulation OR transcranial direct current stimulation OR tDCS OR neurofeedback OR deep brain stimulation). The reference lists of relevant papers were inspected for further studies that fit the inclusion criteria. The search was conducted on the literature before August 2017 without historic limitation, and only studies written in English were included. As there are large differences in number, quality and methodologies of studies between the neuromodulation techniques we used specific inclusion criteria to identify the highest quality of available evidence for each technique where we prioritize (1) sham-controlled designs (2) outcome measures focused on clinical efficacy (3) number of sessions for non-chronic neuromodulation techniques (4) and number of participants. Because of the large differences in quality and quantity of studies between the modulation techniques, we will outline which of the criteria will be applied at the beginning of each section below.

## Results

### Non-Invasive Brain Stimulation (TMS & tDCS)

Non-invasive brain stimulation techniques are growing in popularity as they offer a safe, economical and increasingly accessible means of modulating neural activity. The two most common and best developed methods are rTMS and tDCS. The majority of studies investigating rTMS and tDCS for addiction are single session experimental investigations that incorporate a vast array of stimulation parameters, participant characteristics, and outcome measures. The results of these studies are heterogeneous and do little to determine clinical efficacy. Of the small number of trials delivering multi-session stimulation many lack a sham condition and rely only on self-report measures, which is problematic as this does not control for placebo response or desirability reporting. Therefore, we focus exclusively on studies that meet the following criteria:

- Five or more stimulation sessions
- Include a sham stimulation comparator condition,
- Deliver stimulation with the express aim of modifying craving and/or frequency of engaging in addictive behaviors.

We note that a course of >5 stimulation sessions is a substantially lesser dose than the 20–30 sessions typically considered a therapeutic course for psychiatric indications such as major depression. However, as addiction trials have delivered acute courses ranging from 5 to 13 stimulation sessions this threshold is reflective of the current state of clinical research in this area.

### Transcranial Direct Current Stimulation

tDCS involves application of a low voltage (typically 0.5 mA – 2 mA) electrical current to the scalp via surface electrodes. Current flows in one direction from a positive anode electrode to a negative cathode electrode. There are many research trials investigating tDCS, likely because of its ease of administration, low side effect profile and low cost. The strength and direction of neuromodulation effects are determined by the density, duration and direction of the current that penetrates the skull and comes into contact with underlying neurons (Batsikadze et al. 2013; Jamil et al. 2017; Rawiji et al. 2017). The impact of tDCS on neuronal activity arises from two mechanisms: 1. initial sub-threshold depolarization or hyperpolarization of neuronal membrane potentials which increases or decreases (respectively) the likelihood of spontaneous neuronal firing and 2. (following prolonged / repeated stimulation) facilitation of long-term potentiation or long-term depression like synaptic plasticity (Woods et al. 2016). Proposedly anodal stimulation enhances cortical excitability via depolarization and long-term potentiation whereas cathodal stimulation inhibits excitability via hyperpolarization and long-term depression (Nitsche and Paulus 2000). It is noteworthy, however, that the effects of anodal and cathodal stimulation vary considerably depending on stimulation protocols and inter-individual differences in neurophysiology, clinical status and cognitive capacity (Batsikadze et al. 2013; Jamil et al. 2017; Strube et al. 2016).

tDCS for addictions has been investigated in 7 sham-controlled trials. All stimulated the dorsolateral prefrontal cortex (DLPFC) (see Table 1). Four investigated bilateral DLPFC stimulation, two manipulated current density and flow to favor the left DLPFC and one to the right (i.e. by applying a larger or extra-cephalic return electrode). The rationale for stimulating the DLPFC is based on three observations: 1. The DLPFC has been implicated in spontaneous and cue elicited craving, 2. The DLPFC is involved in decision making, inhibitory control, attentional bias, and awareness, 3. Stimulation of the DLPFC may affect the reward circuitry via efferents to the nucleus accumbens (NA) and ventral tegmental area (VTA).

**Table 1:** tDCS studies for addictiontable

Publication	Addiction	Concurrent Treatment	n	Target	Anode	Cathode	Current density mA/cm	No. Sessions	Objective Outcome Measure	Primary Outcome Measure	Mean % change
Boggio et al. 2009	nicotine	NR	27	DLPFC	F3, 35 cms2	F4, 100 cms2	0.06	5	no	Cue-elicited craving	NI
											At last session: Active: 2.6
											Sham = 4.18
											SD
Fecteau et al. 2014	nicotine	NR	12	DLPFC	F4, 35 cms2	F3, 35cms2	0.06	5	Expired CO2, ultimatum game and risk task	Expired CO2	NI
											NS
da Silva et al. 2013	alcohol	Yes	13	DLPFC	F3, 35 cms2	R supradeltoid, 35 cms2	0.06	5	ERPs to alcohol and neutral cue exposure with LORETA	Relapse	Active: 67% RE
											Sham: 14% RE
											NS
Klauss et al. 2014	alcohol	Yes	35	DLPFC	F4, 35 cms2	F3, 35cms2	0.06	10 (2 per day)	FAB	Relapse	At last session: NI
											NS
											Relapse at 6 months
											Active: 50% RE
											Sham: 88% RE
											SD
Conti and Nakamura-Palacios 2014	crack cocaine	NR	13	DLPFC	F4, 35 cms2	F3, 35cms2	0.06	5	ERPs to cue exposure with LORETA	N2 component in ACC during crack pics	NI
											SD
Batista et al. 2015	crack cocaine	NR	17 pts., 19 controls	DLPFC	F4, 35 cms2	F3, 35cms2	0.06	5	no (urine samples showed no use during study - in rehab)	Craving	NI
											SD
Ljubisavljevic et al. 2016	food	NR	27		F4, 35 cms2	L supraorbit, 35 cms2	0.06		no	Craving*	Active: 14%
										FCQ-T	Sham: 5%
											SD

*NR*, not reported; *NI*, pre and post mean data not included; *SD*, significantly different; *NS*, no significant difference found; *DLPFC*, dorsolateral prefrontal cortex; *LORETA*, low-resolution brain electromagnetic tomography; *FAB*, Frontal Assessment Battery; *FCQ-T*, Food Craving Questionnaire - Trait: *, primary outcome measure not specified; *RE*, relapse

Two controlled trials have investigated the impact of tDCS on cigarette craving and consumption (Table 1). Boggio et al. (2009) used a cross-over design to compare craving in response to visual smoking cues and cigarette consumption during a course of five consecutive daily sessions of left DLPFC anodal tDCS. Active stimulation was associated with greater reduction in self-reported craving intensity and number of cigarettes consumed (median 30% reduction in cigarettes smoked during active tDCS versus 10% reduction during sham), however the integrity of stimulation blinding was not assessed, and no objective outcome measures used. Fecteau et al. (2014) focused on aspects of decision making that contribute to smoking behaviors. Also using a five-session (over 5 consecutive days) cross-over design and cue-reactivity paradigm they found that, relative to sham, bilateral DLPFC tDCS reduced number of cigarettes consumed by 3–4 per day, decreased reported desire to smoke in response to cues, but did not modulate other aspects of craving. Moreover, trend level reduction in exhaled carbon monoxide was observed. Interestingly, using the Ultimatum Game this study found that participants rejected more offers of cigarettes following active tDCS whereas responses to monetary offers were unchanged indicating that motivation for cigarettes changed while preserving motivation for non-drug rewards.

Two tDCS trials conducted in alcohol dependence both delivered stimulation in addition to routine outpatient treatment (Table 1). They differed in tDCS protocol and therapeutic outcomes. Klauss et al. (2014) tested bilateral DLPFC tDCS and applied two short stimulation sessions separated by 20-min interval (a protocol shown to induce prolonged excitatory after effects in the motor cortex; Monte-Silva et al. 2013) across five consecutive days. Both active and sham tDCS were associated with reduced craving for alcohol, mild decrease in anxiety and increase in DLPFC mediated cognitive functions assessed by the Frontal Assessment Battery (FAB). At six-month follow up, however, a significantly lower relapse rate was reported for participants who had undergone active tDCS, with 50% having returned to regular heavy drinking compared to 88% in the sham condition. In contrast, da Silva et al. (2013) applied one session of active vs sham left anodal tDCS per week for five weeks and monitored participants for four further weeks post-stimulation. Active tDCS was associated with acute reduction in craving, depressive severity and density of prefrontal event-related potential responses to both alcohol-related and neutral visual cues, and tDCS did not impact anxiety or performance on the FAB. Sixty-seven percent of participants in the active condition and 14% in the sham condition relapsed during the program. While this pattern appears suggestive of an iatrogenic effect of active tDCS, given the infrequent stimulation schedule it is unlikely that this is the case. The effects of a single tDCS session wear off within hours, making the cumulative impact of one tDCS session per week negligible (Monte-Silva et al. 2013). Indeed, experimental tDCS studies typically use between session wash-out periods of one week, and as such it is difficult to attribute either positive or negative observations in this study to tDCS (Stagg and Nitsche 2011; Woods et al. 2016).

The impact of tDCS on addiction to crack cocaine has been studied in two controlled trials, both of which delivered right anodal and left cathodal DLPFC stimulation (Table 1). Batista et al. (2015) applied active or sham tDCS every second day for ten days. While active stimulation was associated with reduction of craving, depression and anxiety, the efficacy of tDCS blinding was not reported and the study only used subjective outcome measures. Conversely, Conti and Nakamura-Palacios (2014) performed one of the few trials to include a biological outcome measure: following five consecutive daily stimulation sessions there was a significant decrease in anterior cingulate cortex reactivity during exposure to crack cocaine cues following active but not sham tDCS.

There is a dearth of research into tDCS for behavioral addictions. Only a single trial has been conducted into food craving in healthy individuals, specifically excluding those with a history of binge eating or other eating disorder, which did not report on changes in eating behavior (Ljubisavljevic et al. 2016; Table 1). Significant reduction in food craving was documented after an initial active session of right anodal DLPFC tDCS, with superior outcome for active relative to sham tDCS maintained following four further tDCS sessions and at 30-day follow up. Integrity of the sham control was verified by asking participants to guess their stimulation condition at the end of the tDCS course, and a majority (>85%) in both conditions believed they received active stimulation.

Overall, the results of controlled trials of tDCS for addiction are inconclusive. While modest positive effects on craving have been reported by a number of studies findings are inconsistent and the short courses of DLPFC stimulation applied to date have not been associated with prominent or persistent behavioral change. In order to evaluate the potential of tDCS for addiction, trials delivering longer courses of stimulation, prioritizing long-term outcomes and including objective neurophysiological and cognitive measures are required.

#### Cognitive Candidates for tDCS

Because of its subtle and diffuse effects tDCS is poorly suited to target specific aspects of cognition. It is plausible that prefrontal tDCS could alter processing in numerous addiction related cognitive domains, including risky decision making, cognitive control, response inhibition, sensitivity to reward/punishment, and attentional bias. While a number of addiction studies have documented transient modulation in these domains (Fecteau et al. 2014; Nakamura-Palacios et al. 2016), others have not (T E den Uyl et al. 2015; Klauss et al. 2014). It is noteworthy that a recent large (*n* = 200) well controlled investigation into the impact of tDCS on selective attention and risky decision making in healthy individuals failed to replicate the prior positive findings of smaller tDCS studies (Russo et al. 2017). While it is possible that a ceiling effect may have contributed to this null finding, as healthy individuals do not have pathological cognitive or neural processes to modify, it does highlight the need for caution when extrapolating the results of the many studies that report evidence of tDCS induced cognitive modulation. Overall, the findings from the broad tDCS cognitive neuroscience literature have provided little to no evidence of reliable or substantial modulation in any cognitive domain. It has, however, highlighted significant inter-individual differences in response to tDCS (Strube et al. 2016; Wiethoff et al. 2014) and it may be that an individualized approach of delivering stimulation specifically to patients who are physiologically responsive to it and show a cognitive phenotype related to abnormalities in the prefrontal cortex could be a way forward. Finally, the impact of repeated stimulation sessions, concurrent addiction therapies, and tDCS protocols requires systematic study, and the potential of focal high definition-tDCS montages (HD-tDCS) that use a greater number of stimulating electrodes to induce more focal and robust neurophysiological effects is worthy of investigation (Hill et al. 2017).

### Transcranial Magnetic Stimulation for Addictions

rTMS involves application of a time-varying magnetic field to a target region of the scalp via a stimulating coil. The magnetic field penetrates the skull and induces a focal electrical current which depolarizes underlying cortical neurons. Unlike tDCS, the strength of rTMS pulses are sufficient to induce action potentials. The direction and duration of effects are governed by the intensity, number, properties and pattern of applied stimulation pulses. High-frequency rTMS (>5 Hz) tends to have excitatory effects and is applied with intention of up regulating activity in the targeted brain region, whereas low-frequency rTMS (< 2 Hz) tends to have inhibitory effects and is applied with the intention of down regulating activity in the targeted region (Chen et al. 1997; Siebner et al. 2000) although as with tDCS individual variations in response can be substantial (Maeda et al. 2000). Several types of TMS coils have been developed which differ in the focality and penetrative depth of the stimulation. rTMS for addiction trials have used both figure-of-eight coils, which produce highly focal stimulation in superficial cortex, and H-coils, known as deep-TMS, which are designed to achieve deeper intracranial penetration and stimulate a broader region of tissue extending from the cortical surface through to a more ventral target (Deng et al. 2013).

Using the above mentioned criteria, we found 9 studies that investigate rTMS as addiction intervention (see Table 2). As with tDCS, a majority of rTMS trials (six of nine) have targeted the DLPFC by using figure-of-eight coils. Using the H-coil two studies have additionally tested stimulation of the insular and medial prefrontal cortex. The insula is a critical mediator of decision-making that involves weighing the pleasurable interoceptive effects of substance use and other addictive behaviors against their potential negative consequences (Naqvi and Bechara 2010). The medial prefrontal cortex is involved in a myriad of decision making processes (Euston et al. 2012), and plays a particularly important role in (mal)adaptive responses to stressful and rewarding events and, via connections to the hypothalamic pituitary adrenal axis, ventral tegmental area and the nucleus accumbens, may offer a more direct pathway than the DLPFC to neural circuits linked to motivation control, reward and pleasure. As addiction involves dysfunction in multiple neural circuits the ability to non-invasively modulate both cortical and subcortical brain regions makes rTMS an appealing clinical tool for this indication.

**Table 2:** TMS studies for addictiontable

Publication	Addiction	Concurrent Treatment	rTMS Coil	Sham Condition	n	Target	Localisation	Hz	No. sessions	Pulses per session	% RMT	Objective Outcome Measure	Primary Outcome Measure	Mean % change in Primary Outcome Measure
Amiaz et al. 2009	Nicotine	NR	Figure of eight	Mu-metal plate coil shield	48	L DLPFC	5-cm method	10	10 a + 6 m	1000	100	Urine (cot/cre ratio)	Urine	Active:50%
														Sham: <1%
														SD
Trojak et al. 2015	Nicotine	Nicotine replacement therapy	Figure of eight	Sham coil	37	R DLPFC	MRI-guided neuronavigation	1	10	360	120	Expired carbon dioxide conc.	Abstinence	Active: 89% AB
														Sham: 50% AB
														SD
Höppner et al. 2011	Alcohol	NR	Figure of eight	Tilted +5cms lateral to F3 + 60% MT	19	L DLPFC	F3	20	10	1000	90	Attentional blink	Craving (OCDS)	NI
														NS
Su et al. 2017	Meth.	NR	Figure of eight	Tilted	30	L DLPFC	5-cm method	10	5	1200	80	Cognitive battery	Craving	Active: 79%
														Sham: 35%
														SD
Gay et al. 2016	Food	No	Figure of eight	Sham	47	L DLPFC	6-cm method	10	10	1000	110	no	Binge episodes	Active: 30%
														Sham: 52%
														NS
Walpoth et al. 2008	Food	No	Figure of eight	Sham	14	L DLPFC	NR	20	15	2000	120	no	*Binges per day	Active: 6%
														Sham: 40%
														NS
Dinur-Klein et al. 2014	Nicotine	NR	H-coil	Sham	115	B DLPFC + insular	dTMS: 6-cm anterior to MTS	1, 10, sham dTMS	13	high: 990 Low: 600	120	urine analysis (cot/cre ratio)	Urine	NI
														SD
Ceccanti et al. 2015	Alcohol	No	H-coil	Active/sham device activation card	18	medial PFC	dTMS: 5-cm anterior to MTS	20 dTMS	10	1500	120	blood cortisol and blood prolactin	Alcohol intake on max intake days	Active: 100%
														Sham: 76%
														NS
Bolloni et al. 2016	Cocaine	NR	H-coil	Sham	10	B PFC	dTMS: not described	10 dTMS	12	1000	120	hair analysis	Hair	NI
														NS

*NR*, not reported; *NI*: pre and post mean data not included; *SD*, significantly different; *NS*, no significant difference found; *L DLPFC*, left dorsolateral prefrontal cortex; *R DLPFC*, right dorsolateral prefrontal cortex; *B DLPFC*, bilateral dorsolateral prefrontal cortex; *medial PFC*, medial prefrontal cortex; *B PFC*, bilateral prefrontal cortex; *OCDS*, obsessive compulsive drinking scale; *AB*, abstinent; *RMT*, resting motor threshold;*, primary outcome measure not specified

The two rTMS trials for nicotine addiction have stimulated the DLPFC, and both reported positive but short-lived outcomes (Table 2). Amiaz et al. (2009) delivered 10 sessions of high-frequency left DLPFC rTMS followed by a maintenance series of six sessions over the subsequent month. While a prominent placebo effect on self-reported cigarette consumption was noted, relative to sham, active rTMS was associated with fewer number of cigarettes smoked, lower nicotine consumption (verified via urine analysis), and reduced cue-induced craving. Beneficial effects, however, were largely lost at six-month follow up. Trojak et al. (2015) targeted the right DLPFC and delivered 10 sessions of low-frequency rTMS throughout the first two weeks of a six-week course of nicotine replacement therapy. Both active and sham rTMS groups reported reduced craving, active rTMS was associated with greater maintenance of abstinence throughout the program (verified via exhaled carbon monoxide analysis), but beneficial effects were no longer evident at six-week follow up.

The largest study of rTMS for addiction is particularly interesting because it is the only study that targeted multiple brain regions and compared alternative stimulation protocols (Table 2). Dinur-Klein et al. (2014) used the H-coil to stimulate bilateral ventrolateral prefrontal and insular cortices and compared high and low frequency rTMS on craving and cigarette consumption. Additionally, they tested the effect of cue exposure before stimulation. Following 13 sessions administered over three weeks, cue-exposure did not substantially influence outcomes and craving did not differ between the groups, but greatest reduction in number of cigarettes consumed per day (self-report and urine analysis) and highest abstinence rates at end of treatment were observed following high-frequency rTMS.

It is notable that, as with a number of the studies outlined above, a number of rTMS trials for alcohol addiction show a therapeutic response with sham treatment, which highlights the necessity of robust sham conditions and evaluation of participant blinding, and reporting of whether or not concurrent addiction therapies are being undertaken by participants. Höppner et al. (2011) reported that, relative to sham stimulation, 10 sessions of high frequency left DLPFC rTMS increased the attentional blink effect to (i.e. reduced preconscious salience of) alcohol-related pictures, but all participants reported reduced craving for alcohol and improved mood irrespective of stimulation condition. Reduction in the amount of alcohol consumed per day in response to both active and sham stimulation was also described by Ceccanti et al. (2015), though active rTMS was specifically associated with reduced craving. Participants underwent 10 sessions of medial prefrontal cortex H-coil stimulation paired with olfactory drink-of-preference cues. However, the application of only within group analyses (i.e., no direct comparisons between outcomes of active and sham TMS) and high dropout rate during the follow up assessment period limit conclusions that can be drawn from the results.

The only other investigation to encompass medial prefrontal cortex stimulation was conducted by Bolloni et al. (2016) in a cohort of participants with cocaine addiction (Table 2). High frequency bilateral prefrontal deep-TMS was applied three times per week for four weeks and a significant reduction in cocaine use, as indexed by hair toxicology, observed following both active and sham stimulation. When the two groups were analyzed separately, however, persistent reduction in cocaine use three and six months post treatment were significant only for participants who underwent active rTMS.

The single trial of rTMS for methamphetamine addiction is also the only controlled trial to include cognitive outcome measures, albeit largely cognitive measures that are indirectly implicated in addiction decision making (e.g., error monitoring or working memory). Su et al. (2017) delivered five daily sessions of high frequency left DLPFC rTMS as part of a mandatory addiction treatment program. They found a reduction in the intensity of visual drug-cue induced craving in active relative to sham stimulation, and reduction in already low depression severity in both sham and active stimulation, and no differences in anxiety scores in either condition. Across cognitive measures a small improvement in verbal learning and emotional facial recognition following active rTMS was reported, while working memory, problem solving and error monitoring, and visuospatial memory were unchanged.

There is a lack of controlled trials investigating rTMS for behavioral addictions. Two trials have investigated rTMS for bulimia nervosa (as standalone treatment), often conceptualized as one of the numerous presentations of food addiction, and neither found it to be effective beyond sham stimulation. Gay et al. (2016) did not see any beneficial effect on number of binge eating episodes or pre-binge food craving following 10 sessions of active or sham high frequency left DLPFC rTMS. Following 15 sessions of high frequency left DLPFC rTMS, Walpoth et al. (2008) observed significant improvements in binge frequency, obsessive-compulsive and depression symptoms in both the active and sham stimulation conditions.

In summary, the state of evidence for rTMS for addictions is similar to that of tDCS, with a number of promising findings reported alongside numerous negative and inconstant outcomes. The diversity in outcomes likely stems from short treatment courses, variable rTMS protocols and participant characteristics, small sample sizes, and frequent use of subjective and/or short-term outcome measures (Table 2). It is noteworthy that while most rTMS addiction trials have used sham TMS coils, the most robust method for participant blinding, many have observed prominent benefit following sham stimulation. As many studies do not specify the treatment status of participants (see Table 2), apparent placebo response may reflect response to a concurrent treatment regimen. To clarify this issue, future studies should control for and report the treatment status of patients during rTMS treatment and report the efficacy of participant blinding.

#### Cognitive Candidates for rTMS

As a depolarizing technique that can induce focal or deep stimulation, rTMS is somewhat better placed than tDCS to target specific neural circuits and thereby specific aspects of cognitions. There are a few rTMS studies in addiction that have additionally investigated its effect on different aspects of impulsivity. Impulsivity has been associated with vulnerability for addiction (Allen et al. 1998; Diergaarde et al. 2008). Several studies looked at choice impulsivity and there are indications that rTMS can affect delay discounting in smokers, but the results are ambiguous, and clarification awaits further studies using consistent delay discounting paradigms and examining the impact of rTMS parameters (Bickel et al. 2017; Sheffer et al. 2013; Zack et al. 2016). Moreover, no effects above sham stimulation were found on motor impulsivity (go/no-go task) using a single right DLPFC rTMS session in patients addicted to alcohol (Herremans et al. 2013). A more promising avenue might be to use theta burst TMS that more closely mimics the intrinsic neural oscillations that support cognitive processing (Bickel et al. 2017; Zack et al. 2016). In addition, precision targeting methods such as fMRI guided targeting of cortical regions with strong functional connectivity to subcortical targets of interest have both shown potent cognitive effects (Verbruggen et al. 2010; Wang and Voss 2015). This latter method is a particularly promising avenue to modulate key subcortical nodes within the addiction decision making neurocircuitry, such as the ACC or NA.

### Neurofeedback

With neurofeedback, the brain is not directly stimulated as with the previous interventions, but patients learn to modulate their own brain activity through feedback. The brain activity of a patient is monitored and when it shows either the desired or unwanted qualities the patient is informed though visual feedback (e.g., a thermostat that increases when more activity in the desired frequency or brain region) or auditory feedback. Over time the patient will then develop strategies to increase the desired brain activity or decrease the unwanted brain activity. It is therefore a less direct way of neuromodulation that involves training of the participant. Because the two types of neurofeedback that have been used to treat addiction, fMRI and EEG neurofeedback, differ much in the duration they have been used, the number of studies available, the approach (used as stand-alone or add-on treatment), and design, we will use separate criteria for inclusion.

For EEG neurofeedback many studies, case reports and case series have been published but the vast majority is of poor quality: small sample sizes, few training sessions, lack of control condition. We exclusively on studies that meet the following criteria:

- Minimal sample size of 10 per group
- 10 or more EEG neurofeedback sessions
- Inclusion of a control condition without neurofeedback

In contrast to EEG neurofeedback the application of fMRI neurofeedback as intervention for addiction is very new. The first studies were published in 2013 and are mostly proof of principle studies with the aim of investigating (1) whether participants can modulate the targeted brain activity (2) and whether this affects craving. No studies to date have investigated the effects of fMRI on drug use; have used sample sizes larger than 15; or used more than 3 fMRI neurofeedback visits. Moreover, only three studies used a control group (Hartwell et al. 2016; Karch et al. 2015; Kirsch et al. 2016). Since the limited total number of studies on the effect of fMRI neurofeedback on craving, we will not use exclusion criteria. However, we will take this lack of criteria with us in the final evaluation of the different techniques in the discussion.

### EEG Neurofeedback

The oldest method of neurofeedback is EEG neurofeedback. Electroencephalography (EEG) is a neuroimaging method that records electric activity of the brain through electrodes placed along the scalp. It can measure neural oscillations in specific frequency domains that are associated with functions such as attention or relaxation. With EEG-neurofeedback participants can be trained to amplify or decrease oscillations in certain frequency bands. For instance, in patients with Attention Deficit Hyperactivity Disorder EEG neurofeedback is often used to decrease theta oscillations that are related to relaxation and to amplify beta oscillations associated with concentration (Butnik 2005).

With our search we found only seven studies that investigated EEG neurofeedback as treatment for substance and non-substance related addiction (i.e., used an addiction related outcome measure) (see Table 3).

**Table 3:** EEG neurofeedback studies for addictiontable

Publication	Addiction	Concurrent Treatment	n	Target	Control	No. Sessions	Addiction outcome	Other outcome	Follow-up time
Peniston and Kulkosky 1989	Alcohol	Inpatient rehabilitation treatment	10/10/10	Alpha-theta training	(1) treatment as usual	15	Decreased relapse after 13 months follow up	Reduced depressive symptoms	13 months
					(2) healthy controls				
Arani et al. 2010	opioid	Medication	10/10	Alpha-theta training	(1) treatment as usual	30	Decreased craving	Reduced hypochondriacs, obsession, interpersonal sensitivity, aggression, psychosis, and general symptomatic indexes on SCL-90	NR
Schmidt and Martin 2016	Binge eating	NR	18/18/21	Adapting Beta activity after cue exposure	(1) mental imagery	10	Reduced frequency of binge eating and food craving compared to waitlist	Beneficial effects on perceived stress and dietary self-efficacy compared to waitlist	3 months (effects remained stable)
					(2) waitlist				
Rostami and Dehghani-Arani 2015	Crystal Meth	Medication	50/50	SMR and alpha/theta training	(1) treatment as usual	30	Improved addiction severity	Improvement in mental health and quality of life	NR
Dehghani-arani et al. 2013	opioid	Medication	10/10	SMR and alpha/theta training	(1) treatment as usual	30	Decreased craving	Improvements in somatic symptoms, depression and general health	NR
Scott et al. 2005	Mixed substances	Inpatient rehabilitation treatment	60/60	Beta, SMR and alpha-theta training	(1) additional counseling sessions time-matched	40–50	Patients stayed longer in treatment	Improvements in commissions (impulsivity and variability) trend in omission (inattention)	12 months (more abstinence in experimental group)
Lackner et al. 2016	alcohol	Inpatient rehabilitation treatment	12/13	Alpha-theta training	(1) treatment as usual	12	No difference on craving	No difference on depression	5 months only with the 6/7 patients still in clinic and found no difference

*NR*, not reported; *NI*, pre and post mean data not included; *SD*, significantly different; *NS*, no significant difference found; *DLPFC*, dorsolateral prefrontal cortex; *LORETA*, low-resolution brain electromagnetic tomography; *FAB*, Frontal Assessment Battery; *FCQ-T*, Food Craving Questionnaire - Trait; *, primary outcome measure not specified; *RE*, relapse

The first EEG neurofeedback treatment studies for addiction go back to the seventies and focused on training participants to modulate alpha oscillations, which would improve their ability to relax. The underlying idea was that this training could have a therapeutic effect on the addicted individuals either directly or via secondary improvements such as anxiety reduction (Sokhadze et al. 2008). Yet the results on addiction related outcome measures were disappointing (Jones and Holmes 1976; Passini et al. 1977). At the end of the eighties Peniston and Kulkosky popularized the use of EEG neurofeedback as treatment for addiction with a new protocol for alcohol addicted patients that modulated both alpha (8–13 Hz) and theta (4–8 Hz) frequencies in order to bring patients in a relaxed or meditative state (alpha-theta protocol). About ten years later this protocol was adapted by Scott et al. (2005) who expanded alpha-theta training, with beta (16-21 Hz) or sensorimotor rhythm (SMR) (12–15 Hz) training, which aims to improve inhibition. This protocol was thought to be more suitable for patients with other addictions than alcohol, specifically stimulant addiction. The alpha-theta and the alpha-theta augmented with SMR training are now the two main protocols used for treating addiction with EEG neurofeedback (see Table 3).

The Peniston and Kulkosky study (1989) developed the alpha-theta protocol and found more sustained prevention of relapse (defined as not using alcohol for more than 6 contiguous days) in 13-months follow up in alcohol addicted patients who were treated with EEG neurofeedback as add-on treatment compared to patients who only received standard treatment. However, a later study by Lackner et al. (2016) who also used the theta-alpha protocol found no effects on craving or on the 5-month follow up assessment in a group of alcohol addicted in-clinic patients. This same protocol was used in opioid users add-on to psychopharmacological treatment, where they found reduced craving compared to the group that only received medication (Arani et al. 2010).

The combined alpha-theta and SMR training developed by Scott et al. (2005) was first investigated as an add-on treatment for standard rehabilitation program, where they observed longer treatment compliance, and a higher percentage of abstinence (77% of experimental group compared to 44% of control group) at 12 months follow up compared to a control group that received additional counseling (matched in time to neurofeedback sessions). Two more studies used SMR-training in addition to theta-alpha protocol to treat methamphetamine (Rostami and Dehghani-Arani 2015) or opioid (Dehghani-Arani et al. 2013) addiction and found decreased severity of addiction and reductions in craving compared to the treatment as usual (pharmacotherapy) control group. Neither study included a follow up assessment. Finally, the last study looked at a subclinical group of women with binge eating episodes but is interesting to mention in the light of the broader non-substance addiction perspective. They used a different protocol that aims to reduce high beta activity (23–28 Hz) and found reduced frequency of binge eating that remained stable over a 3-month follow up (Schmidt and Martin 2016).

Considering the years that EEG neurofeedback has been studied the low number of studies of reasonable quality is disappointing, which suggests a lack of interest of the field for a well-established efficacy. Even the above discussed studies use a wide variety of outcome assessments which makes the results hard to compare and only one study used a control condition matched in time (Scott et al. 2005). The SMR alpha-theta protocol showed the most promising results for the treatment of addiction but more systematic study is needed to establish for which patients it is most effective, the impact of concurrent therapy, and the optimal protocol.

#### Cognitive Candidates for EEG Neurofeedback

Two studies (additionally) investigated the effect of mixed SMR theta-alpha EEG neurofeedback on motor-impulsivity in a group with mixed-substance abuse as measured by a cognitive go/no-go task (Keith et al. 2015; Scott et al. 2005). They both found a reduction in commission errors (i.e., responses to no-go trials) in the group that received neurofeedback compared to the control group (with alternative therapy). This suggests the mixed SMR alpha-theta protocol could be specifically effective for treating addiction in high impulsive patients.

### fMRI Neurofeedback

Functional Magnetic Resonance Imaging (fMRI) measures increases or decreases of blood oxygenation level dependent (BOLD) response in areas of the brain indicating increased or decreased activity of that area. The main advantage of fMRI over EEG is that it can cover the whole brain (including subcortical areas) and has a much higher spatial resolution. However, preprocessing and analysis of fMRI data used to take much time and thereby made it unsuitable for direct feedback. This changed with the invention of real-time fMRI, where the data are analyzed during collection enabling direct feedback (deCharms 2008). With fMRI neurofeedback it is possible to provide feedback about the activity of one region, a combination of regions or about the functional connectivity between regions. This feedback is mostly visually presented to participants for instance by a thermostat that moves up or down when the targeted activity or connectivity increases or decreases. Therefore, fMRI neurofeedback makes it possible to train people to modulate the activity of specific brain regions or connections.

We found 8 studies that investigated the effects of fMRI neurofeedback on craving (see Table 4).

**Table 4:** fMRI neurofeedback studies for addictiontable

Publication	Addiction	Concurrent Treatment	n	Target	ROI selection	Control	No. Sessions	Addiction outcome
Hartwell et al. 2013	Nicotine	No	10 FB	[1] Reduce craving by decreasing ACC activity	Individual: Selected while viewing nicotine related pictures	No	1	[1] Reduced subjective craving and [2] reduced activation in ACC condition [3] ACC activity was correlated with craving [4] No differences in mPFC condition
				[2] Increase resist the urge to smoke by increasing mPFC activity				
Hartwell et al. 2016	Nicotine	No	16 FB	Reduce craving ACC activity	Individual: Selected while viewing nicotine related pictures	(1) No FB (blank thermometer)	3	[1] Reduced subjective craving (in anticipation of positive outcome; no effect in expectation of relief of negative effect) [2] Reduced activation in ROI
			17 C(1)					
Hanlon et al. 2013	Nicotine	No	9–15^1^	[1] Reduce craving by decreasing vACC	Individual: Selected while viewing nicotine related pictures	No	3	[1] Reduced subjective craving [2] reduced activation in ACC condition [3] ACC was correlated with craving reductions [4] No differences in mPFC condition
				[2] Increase resist the urge to smoke by increasing dmPFC activity				
Kirsch et al. 2016	Alcohol (heavy drinking students)	No	13 FB 13 C(1) 12 C(2)	Reduce activity in VS that was selected with reward paradigm	Individual: selected with reward paradigm	(1) Yoke (other participant) (2) No FB	1	[1] No increase of craving in FB and C(1) compared to C(2) condition [2] stronger decline of striatal acitivation in FB compared to control groups [3] stronger activation in IFG in FB compared to controls that correlated with VS reduction [4] No correlation between subjective craving and VS activation in any group
Karch et al. 2015	Alcohol (heavy drinking students)	No	13 FB	Reduce craving by decreasing activity in the ROI (selected from most reactive region between ACC DLPFC or insula)	Individual: Selected while viewing alcohol related pictures	(1) HC with FB	1	[1] Within patient FB group reduced craving found, no effects in control groups (no direct comparisons with control groups reported) [2] Reduced activity in ROI in patient and control FB, no effect in sham-feedback (no direct comparisons between FB and sham conditions reported)
			14 C(1)					
			2 C(2)					
			5 C(3)			(2) AP with yoke (other region)		
						(3) HC with yoke (other region)		
Li et al. 2013	Nicotine	No	10	[1] Reduce craving by decreasing ACC activity	Individual: Selected while viewing nicotine related pictures	No	1	[1] Reduced subjective craving [2] reduced activation in ACC condition [3] ACC was correlated with craving reductions [4] No differences in mPFC condition
				[2] Increase resist the urge to smoke by increasing mPFC activity				
Canterberry et al. 2013	Nicotine	No	9	Reduce craving by decreasing ACC activity	Individual: Selected while viewing nicotine related pictures	No	3	[1] Only effects found in ACC activity between craving condition and NF condition, no effect of visit
Kim et al. 2015	Nicotine	No	7 FB(1)	FB(1) reduction of combination of bilateral ACC mPFC and OFC activity; FB(2) above described activity combined with FC between posterior and anterior craving regions	From predefined regions in anatomical atlas	No but 2 FB groups for comparison	2	[1] Greater neural activity and increased FC in FB(2) compared to FB(1) [2] lower craving in FB(2) [3] In FB(2) neural activity and FC were associated with craving but not in FB(1)
			7 FB(2)					

*FB*, feedback

*mPFC*, medial prefrontal cortex; *OFC*, orbital frontal cortex; *(v)ACC*, (ventral) anterieur cingulate cortex; *ROI*, region of interest; *dmPFC*, dorsomedial prefrontal cortex; *IFG*, inferior frontal gyrus; *DLPFC*, dorsolateral prefrontal cortex; *NF*, neurofeedback

^1^only 60% (9 participants) completed all session, the nr of participants included in the analysis are not specified

In total six studies have investigated the effects of fMRI neurofeedback on nicotine addiction (Canterberry et al. 2013; Hanlon et al. 2013; Hartwell et al. 2013, 2016; Kim et al. 2015; Li et al. 2013). Five of these studies showed that modulating activity from ACC with fMRI neurofeedback while watching smoke related pictures was effective in reducing ACC activity and craving (Canterberry et al. 2013; Hanlon et al. 2013; Hartwell et al. 2013, 2016; Li et al. 2013). Yet, when they were instructed to resist craving and received feedback from the (dorso)medial PFC it did not affect brain activity or craving (Hanlon et al. 2013; Hartwell et al. 2016; Li et al. 2013). The fifth study compared feedback from different craving-related regions of interest (ACC, mPFC and OFC) with feedback that additionally gave information about the functional connectivity between posterior and anterior craving-related regions. They found a stronger effect on brain activity and craving, and a correlation between those two, in the condition with additional functional connectivity information (Kim et al. 2015).

The first two studies on fMRI neurofeedback in alcohol addiction (Karch et al. 2015; Kirsch et al. 2016) indicate that patients are capable of reducing craving related activity in the ACC, PFC, insula (Karch et al. 2015), or in the ventral striatum (Kirsch et al. 2016) (see Table 4). In the latter study the researchers found both in the experimental and sham-control group an increase in PFC activity, which was only related to a reduction in the ventral striatum in the experimental group. Although the study of Karch et al. (2015) did scan a control group, they did not include them in the analysis which makes the interpretation of the results difficult.

In sum, at this moment the evidence for fMRI neurofeedback as intervention for addiction is still scarce and we will need larger controlled trials to establish efficacy on drug use and long-term effects.

#### Cognitive Candidates for fMRI Neurofeedback

Since fMRI is the most commonly used tool to investigate the biological basis of neurocognitive processes related to addiction other than craving, fMRI neurofeedback could be very suitable to target these processes such as reward processing or cognitive control. Hopefully future research will explore these promising avenues.

### Invasive Brain Stimulation: Deep Brain Stimulation (DBS)

In addition to the above mentioned non-invasive techniques, deep brain stimulation (DBS) offers a more direct way for stimulating and modulating the brain. Powered by an implanted pulse generator, DBS provides deeper located brain areas with a continuous stimulation via surgically implanted microelectrodes. The implanted pulse generator, which is located in the pectoral area, can be manually switched on and off from outside the body and furthermore allows for modulation of stimulation frequency and intensity. Originally, DBS was introduced in the 80’s as treatment for intractable movement disorders. However, in the last 15 years it has been successfully applied in psychiatric disorders such as treatment-refractory depression and treatment-refractory obsessive-compulsive disorder (OCD). When studies reported unintended alleviation of comorbid alcohol (Kuhn et al. 2007) and nicotine (Kuhn et al. 2009) dependences following nucleus accumbens (NAc) DBS and reduced pathological gambling (Ardouin et al. 2006) in patients with Parkinson’s disease, DBS gained interest as a tool for addiction treatment.

Since then, several studies have examined the possibility of DBS as a therapeutic tool in addiction treatment. In 2012, Luigjes et al. reviewed possible brain targets for DBS in treatment-refractory addiction based on previously conducted clinical and animal studies. Human studies mainly focused on NAc and subthalamic nucleus (STN) as these areas have been regularly used in other refractory psychiatric disorders. Luigjes et al. (2012) suggested the NAc as most promising target for DBS therapy since both animal and human studies with NAc stimulation showed reduction or cessation of drug intake and long-term abstinence without severe side effects.

The effectiveness of NAc stimulation may be, in part, explained by its central position in brain circuits implicated in motivation and inhibitory control. As a result of repeated drug use the brain circuit implicated in motivational drive could become hyperactive in response to the substance. At the same time, diminished activation of prefrontal areas implicated in regulation of inhibitory processes such as impulse control and decision-making could result in a deficient inhibitory system (Volkow et al. 2013). Combined, these disturbances could result in a heightened drive to obtain and use drugs while it becomes harder to suppress this persistent drive as a result of a deficient inhibitory system.

All studies that investigated the efficacy of DBS for addiction are case reports or case series. To the best of our knowledge, no larger controlled trials have been published. Therefore, we will discuss all the currently available human DBS (case) studies. Currently eight studies in a total of 11 patients have specifically examined the effects of DBS on addiction related behavior (see Table 5). Six out of these eight studies examined unique patients with a treatment resistant course of alcohol, heroin, or cocaine addiction and all but one used the NAc as stimulation target.

**Table 5:** DBS for addiction

Publication	Addiction	Concurrent Treatment	n	Target	Frequency (Hz)	PW (μs)	Voltage (V)	Addiction outcome	Follow-up time	Side-effects
Müller et al. 2009	Alcohol	NR	3	NAc (bilateral)	130	90	3.5–4.5	2 resolved, 1 improved	12–15 months	–
Kuhn et al. 2011	Alcohol	NR	1	NAc (bilateral)	130	90	3.5–4.5	1 resolved	12 months	–
Vöges et al. 2012 *	Alcohol	NR	5*	NAc (bilateral)	130	90	4.5	2 resolved, 3 improved	38 months	–
Müller et al. 2016 **	Alcohol	NR	5**	NAc (bilateral)	130	90	3.5–4.5	2 resolved, 3 improved	4–8 years	1 pat. Experienced hypomanic episode (14 days) after initiation of DBS
Gonçalves-Ferreira et al. 2016	Cocaine	NR	1	Acc (bilateral)	150	150	2.5–4	1 improved	24 months	1 pat. Experienced unpleasant sensation of warmth and metallic taste during optimisation period
Zhou et al. 2011	Heroin	NR	1	NAc (bilateral)	145	90	2.5	1 resolved	6 years	1 pat. Experienced light concusion and incontinence until 12 h post-surgery
Valencia-Alfonso et al. 2012	Heroin	Heroin replacement therapy	1	Nac (bilateral)	180	90	3.5	1 resolved	10 months	–
Kuhn et al. 2014	Heroin	NR	2	NAc (bilateral)	130–140	90–120	4.5–5	2 resolved	24 months	1 pat. Experienced epileptic seizure 2 days post-surgery

Acc, anterior cingulate cortex; Nac, nucleus accumbens; NR, not reported; pat, patient; PW, pulse width *: Three of five patients reported in Vöges et al. (2012) were previously reported in Müller et al. (2009); **: Three out of five patients reported in Müller et al. (2016) were previously reported in Müller (2009), the other two were previously discussed in Vöges et al. (2012)

### DBS Studies

In two studies six patients were treated with bilateral high-frequency NAc DBS for severe alcohol dependence (Table 5). All six patients showed marked reduction of alcohol craving and intake. Two patients, examined in the study of Müller et al. (2016)*,* even remained abstinent during a follow up period of seven years. The patient examined in the study of Kuhn et al. (2011) showed reduction of craving and ceased drinking one year after initial treatment. Additionally, complemented electroencephalography (EEG) measurements in this patient revealed stronger error related activity in the anterior cingulate cortex (ACC) compared to the situation prior to DBS stimulation. This may perhaps indicate that the patient regained cognitive control as result of NAc DBS.

Three studies examined the effect of bilateral high-frequency NAc stimulation in four patients with a chronic treatment resistant course of heroin dependence (Table 5). In the case series of Kuhn et al. (2014) DBS reduced craving and induced heroin abstinence in two treatment resistant patients. Although DBS induced heroin abstinence, both patients reported occasional use of other psychotropic substances such as amphetamine and alcohol. According to the two patients, comorbid substance use resulted from boredom and incapability to cope with occupational strains rather than craving. In the two remaining case reports, one patient remained abstinent and free of craving for the complete follow up period of six years, even when electrodes were first switched-off and explanted after three years (Zhou et al. 2011). The other patient treated with NAc DBS for his heroin dependence reduced his heroin consumption over a period of months and remained abstinent during the 1-year follow up with the exception of a two-week relapse (Valencia-Alfonso et al. 2012).

Finally, a 36-year-old patient with severe refractory cocaine dependence was treated with high-frequency DBS (Table 5). Microelectrodes were implanted in the posterior part of the medial ACC and bed nucleus of stria terminalis. Initiation of DBS after surgery resulted in a considerable reduction of craving and cocaine consumption which was maintained more than 2.5 years post-surgery (Gonçalves-Ferreira et al. 2016).

Although results from the above-mentioned case studies and case reports may seem promising, as of today no double blind controlled DBS trials in addiction are available. It is possible that the clinical application of DBS is regarded as too costly and invasive for the treatment of addiction. Recruitment of sufficiently motivated patients for controlled studies proves difficult (Luigjes et al. 2015). At the same time, recruitment difficulties could be a result of the absence of strong evidence for the effectiveness of DBS and the suitability as technique for addiction, its proven effectiveness in other neuropsychiatric disorders (Denys et al. 2010). Because of this, DBS addiction research seems to have reached an impasse. Therefore, it remains difficult to draw conclusions in terms of effectiveness.

#### Cognitive Candidates for DBS

The lack of controlled double-blind studies shows that DBS addiction research is still in an early stage and the effects of DBS on addiction related cognitive processes remain speculative. Nevertheless, animal and case studies have provided evidence that NAc DBS can modulate impulsivity. Since impulsivity plays an important role in addiction it is a possible future cognitive target candidate (Sesia et al. 2008, 2010). Yet, several human and animal studies indicate that NAc stimulation can modulate impulsivity in both directions. Whether impulsivity is increased or decreased depends on multiple factors including the exact region of stimulation within the NAc (i.e. NAc core or shell), baseline impulsivity levels in animals, stimulus intensity and on which behavioral paradigm was used (Schippers et al. 2017; Sesia et al. 2008, 2010). Additionally, animal studies have observed that DBS differentially affects state impulsivity and trait impulsivity as the effect of DBS could sometimes have a different direction on these two forms of impulsivity (Schippers et al. 2017; Sesia et al. 2010). These studies show that controlled modulation of impulsivity with NAc DBS is very complex and caution is warranted since increased impulsivity may lead to relapse (Dalley et al. 2011). Indeed, such an increase in impulsivity was observed in two refractory OCD patients who were treated with NAc DBS (Luigjes et al. 2011). Therefore, more research is needed to understand how DBS affects impulsivity, and impulsive behavior should be carefully regarded during patient screening and selection in DBS treatment (Luigjes et al. 2011).

### Candidate Targets for Neuromodulation

Several neurocognitive aspects of addiction can also be found in psychiatric disorders more commonly treated with neuromodulation. The effects of neuromodulation on these shared neurocognitive aspects could reveal potential brain modulation targets for addiction. As an example of such neurocognitive overlap, aberrant reward processing is central to addiction but is also found in OCD. Blunted monetary reward anticipation signals in the ventral striatum have been demonstrated in addiction to alcohol, nicotine and cannabis (Bühler et al. 2010; van Hell et al. 2010; Wrase et al. 2007), and in a behavioral addiction like pathological gambling (de de Greck et al. 2010; Reuter et al. 2005). Similarly, patients with OCD display blunted monetary reward anticipation activity in the ventral striatum compared to healthy controls (Figee et al. 2011, 2013). Effective DBS of the ventral anterior limb of the internal capsule (vALIC) in OCD patients is able to normalize these anticipatory reward responses in the ventral striatum (Figee et al. 2013). Of interest, effective DBS for major depression, a disorder that is also associated with ventral striatal reward dysfunction (Pizzagalli et al. 2009), appears to be dependent on stimulation of comparable ventral anterior internal capsule fibers (Lujan et al. 2012). Together, these findings in various disorders of reward dysfunction suggest that DBS targeted at the vALIC may also be effective in addiction via restoration of healthy ventral striatal reward processing. Indeed, vALIC DBS has shown preliminary effectiveness in drug addiction (Valencia-Alfonso et al. 2012).

Addiction and OCD also share a more general dysregulation of the ventral striatum-prefrontal network, in particular frontostriatal functional hyperconnectivity. Studies in OCD demonstrate that this frontostriatal connectivity can be normalized with DBS of the vALIC (Bahramisharif et al. 2016; Figee et al. 2013; Smolders et al. 2013), but also with rTMS of the mPFC (Dunlop et al. 2016). Preliminary evidence suggests that the vALIC and mPFC are also effective targets for DBS and TMS in addiction based on a comparable down-regulation of frontostriatal hyperconnectivity (De Ridder et al. 2011; Figee et al. 2013) Of interest, DBS resulted in attenuated craving for heroin in a patient with heroin addiction when exactly those contact points on the electrode were activated at which the lowest ventral striatal-prefrontal connectivity in response to drug-related pictures was measured (Valencia-Alfonso et al. 2012)^.^ Therefore, down-regulation of ventral frontostriatal connectivity could be examined as a personalized outcome for DBS and rTMS in the treatment of addiction.

Another neurocognitive aspect underlying addiction is an overreliance on negative-reinforcement in anti-reward brain systems. Important structures implicated in these anti-reward systems are the extended amygdala (bed nucleus stria terminalis, BNST) and the lateral habenula (Root et al. 2014), which are associated with aversive or stress-like states. Adaptations of these systems may persist during and beyond drug abstinence, creating a condition of chronic dysphoria and increasing the likelihood of relapse in a compulsive attempt to self-medicate this unwanted condition (Vollstädt-Klein et al. 2010). Negative reinforcement in addiction may be normalized with DBS targeted at brain anti-reward systems such as the BNST or lateral habenula. BNST DBS was reported effective in a patient with refractory cocaine dependence (Gonçalves-Ferreira et al. 2016). Habenula stimulation inhibited cocaine seeking in rats (Friedman et al. 2011).

In addition, medial prefrontal-amygdala circuits involved in fear conditioning and extinction have been implicated in persistent drug-seeking behavior and relapses (Crunelle et al. 2015; Peters et al. 2009). Interestingly, ventral striatal (VS) high frequency DBS in rats, comparable to the settings often used in humans, impaired extinction training of morphine seeking, whereas low frequency dorsal VS DBS strengthened morphine extinction memory (Martínez-Rivera et al. 2016). DBS in this study also modulated activity (c-fos expression) in areas mediating extinction such as the mPFC and amygdala. Thus, low frequency DBS of the dorsal VS might be beneficial for preventing relapses to drug addiction by activating the mPFC-amygdala circuit involved in extinction learning. In line with the potential of mPFC modulation for extinction of addiction-associated negative reinforcement, rTMS of the mPFC was able to reduce craving in alcohol addiction (Ceccanti et al. 2015) and resulted in persistent reduction in cocaine use (Bolloni et al. 2016).

The insula may be another promising neuromodulation candidate for extinction in the prevention of drug relapse. The insula is activated during exposure to drug-associated cues in addicted individuals (Garavan 2010) and its inactivation has been associated with decreased relapse rates of nicotine addiction (Gaznick et al. 2014; Naqvi et al. 2007)^.^ Inhibition of the anterior insular cortex may facilitate extinction of drug seeking after abstinence via modulation of projections to the central amygdala, as was shown in a rat model (Venniro et al. 2017). Although selective neuromodulatory inhibition of the anterior insula has not yet been tried in people with addictive disorders, the insula was recently selectively targeted in healthy individuals with rTMS using an H-coil (Malik et al. 2017). Of interest, low frequency (presumably inhibitory) rTMS of the insula decreased dopamine levels in the substantia nigra and striatum, suggesting that inhibitory neuromodulation of the insula may also influence reward processing.

Finally, it is relevant to note that DBS of the STN is often beneficial for addictive behaviors related to poor impulse control in Parkinson patients. In the majority of cohort studies and case studies, STN DBS diminishes the prevalence of impulse control disorders, such as pathological gambling, shopping, binge eating or dependency towards their dopamine medication, relative to the preoperative situation (Merola et al. 2017). Nevertheless, a reduction of dopaminergic medication after DBS surgery may partly contribute to this improvement of impulse control disorders. Moreover, there have also been measures of impulsivity, such as response inhibition and delay discounting that deteriorated with STN DBS in Parkinson patients (Georgiev et al. 2016). In rats, STN DBS decreases the motivation for cocaine (Rouaud et al. 2010). In parallel to the beneficial effect of STN lesions for addiction (Baunez et al. 2005), STN DBS may be decrease motivation for drug rewards via inhibition of STN excitatory input on the ventral midbrain, resulting in attenuated dopaminergic activity.

## Discussion

At the moment, little evidence supports the hope that neuromodulation will soon become a new effective intervention for addiction. This may be due to a lack of well-powered systematic and controlled studies. In addition, the reviewed studies indicate that it may too simplistic to assume that neuromodulation targets specific brain regions. Non-invasive neurostimulation and EEG neurofeedback are targeted at broader brain regions or networks, as well as DBS, where specific regions are stimulated and distant brain regions are critically involved in the clinical effects. Moreover, due to the heterogeneity of addiction, neurobiological knowledge may not be easily translated into treatment targets. In sum, it is important to realize the complexity of both the neurobiology of addiction and the effects of neuromodulation when applying neuromodulation. And one way forward could be to use study designs that integrate and test more complex models of neuromodulation.

Because of the different number and quality of studies available for each neuromodulation technique we used separate inclusion criteria. This means that we cannot directly compare the efficacy of these studies and therefore we will discuss them separately with respect to the stage of development they are in. The studies with DBS and fMRI neurofeedback are either pilot or proof-of-principle studies that do not warrant any conclusions about the efficacy as addiction interventions. This is not surprising since these interventions as indication for addiction are still new, require a high degree of expertise and are expensive. As Table 4 shows, the fMRI neurofeedback studies are all published in the last five years and have developed over time in terms of quality and samples size, therefore it is to be expected that larger controlled trials investigating the effect on drug use will follow. With DBS, difficulties with the inclusion of patients have hampered larger controlled DBS studies, while such trials have been published for the treatment of depression and obsessive-compulsive disorder. It is unknown whether future developments in the field will overcome these difficulties.

With rTMS and tDCS, most studies have focused on immediate effects of craving. Yet there are a few studies that looked at (immediate and longer-term) effects on drug use, with promising results indicating the potential of these techniques for treating addiction. In this regard, the current state of research is not dissimilar to the early years of experimental rTMS for major depression, which eventually has become an effective treatment. Whether rTMS will ultimately be an effective treatment for addiction as well, awaits larger trials delivering longer treatment courses, incorporating objective and long-term outcome assessments, and systematic investigation of stimulation protocols.

On the other hand, EEG neurofeedback has been investigated for decades and although one of the first studies included a sham feedback control condition (Jones and Holmes 1976), very few studies afterwards have used an appropriate control with either sham-feedback or where at least the time spend on the neurofeedback sessions was matched with another activity. This makes it difficult to draw conclusions about the specific effect of EEG neurofeedback, since any effects might be the result of the extra time and care patients received for the neurofeedback sessions. In one study that did include such a control condition, the control condition was not used as comparison in the final analysis (Schmidt and Martin 2016). Fortunately, there are two exceptions with control condition (time-matched activity) (Keith et al. 2015; Scott et al. 2005) suggesting that the combination of SMR and alpha-theta neurofeedback can have a positive effect on impulsivity, and on the period of time spend in rehabilitation. However, after decades of investigating EEG neurofeedback the lack of well-designed studies is worrisome and raises the question whether this reflects the lack of potential of EEG neurofeedback to treat addiction.

Until now all fMRI neurofeedback studies and most rTMS/tDCS studies have focused on short-term outcome assessments. Yet, in order to apply these neuromodulation techniques as intervention for addiction a crucial question is whether it is possible to bring about lasting changes in well-established addictive behavior. To answer this question, we should not only assess long-term outcomes but also investigate the number of treatment sessions required for these lasting behavioral changes. In most studies short treatment courses were used: fMRI neurofeedback studies (max 3), tDCS (max 10), rTMS (max 16), only in EEG neurofeedback four studies used 30 sessions or more. This might have contributed to the lack of long-term effects found by several studies: Da Silva et al. (2013) with 5 sessions, Amiaz et al. (2009) with 16 sessions, Trojak et al. (2015) with 10 sessions and Lackner (2016) with 12 sessions. It is likely that in order to change neural programs and their associated behavior, extensive practice is needed using more sessions. Therefore, future research in this area should address the dose of neuromodulation requires to achieve persistent long-term reduction in addiction severity.

Moreover, the second main concern of the reviewed studies is that many of them are underpowered. Two fMRI neurofeedback studies (Canterberry et al. 2013; Kim et al. 2015), five rTMS studies (Bolloni et al. 2016; Ceccanti et al. 2015; Dinur-Klein et al. 2014; Höppner et al. 2011; Walpoth et al. 2008) and three tDCS studies (Conti and Nakamura-Palacios 2014; da Silva et al. 2013; Fecteau et al. 2014) used less than 10 participants per condition. Four DBS only case reports or series have been published. This warrants caution with the interpretation of these results, which have a higher change of type II error and inflated effect sizes. However, in all fields except for EEG neurofeedback, increasing the quality of studies for our review by excluding those with less than 10 participants per condition would not have weighed up against the low number of studies left with (0 dB, 6 fMRI neurofeedback, 4 tDCS and 5 TMS). Fortunately in recent years sample sizes are increasing (e.g., Gay et al. 2016; Hartwell et al. 2016; Ljubisavljevic et al. 2016), and in 2017 the largest tDCS trial in addiction was published. In this study, 91 patients were randomized into three groups with the active intervention group receiving a single session tDCS to boost a cognitive bias modification training. No effects of tDCS were found on craving, approach bias or abstinence after three months, but there was a trend towards lower relapse in the tDCS condition after one year (den Uyl et al. 2017). In sum, while there is still a lack of well-powered studies the field seems to be progressing in the right direction and future studies should aim for larger samples sizes.

Neurofeedback, rTMS and tDCS have very few side effects, and are transient; therefore, they are considered safe interventions (Priori et al. 2009). DBS is invasive and side effects such as transient hypomanic episodes have been reported. Additionally, there is a small risk of hemorrhage, infection or headaches. Yet, studies of DBS targeted at the ventral striatal area for other psychiatric disorders have proven to be safe and modulate neurocognitive dysfunctions that are also relevant for addiction (Luigjes et al. 2013). For neuromodulation and non-invasive neurostimulation, future research will have to investigate the number of sessions needed for an optimal effect, and whether patients will benefit from booster sessions after treatment. DBS gives chronic stimulation; however, one case report in a patient treated for opioid addiction showed that the patient remained abstinent even with cessation of stimulation (Zhou et al. 2011).

One question that remains to be investigated is whether neuromodulation techniques can function as stand-alone treatments or whether they are more effective as an add-on treatment. In the majority of EEG neurofeedback studies, the treatment is provided as add-on to either pharmacological treatment or psychotherapy with the rationale that EEG neurofeedback may augment the effects of treatment as usual. Moreover, two tDCS studies used concurrent therapy (de Silva et al. 2003; Klauss et al. 2014) and one rTMS study (Trojak et al. 2015) used additional nicotine replacement treatment. Kuhn et al. (2014) proposed that additional behavioral therapy may be helpful for patients to learn to cope with stress and boredom after DBS treatment which are often triggers for relapse. The combination of DBS and cognitive behavioral therapy has been successful in the treatment of obsessive-compulsive disorder (Mantione et al. 2014). Since addiction is a disorder that affects many aspects of a patient’s life it is likely that additional cognitive behavioral treatment or psychosocial support can play an important role in the success of neuromodulation interventions especially for the most severely affected patients. Alternatively, neuromodulation could enhance the success of add-on treatment. rTMS and DBS have substantial efficacy in mood disorders (Kisely et al. 2018; Perera et al. 2016), and beneficial effects on mood in addiction could enhance motivation for change and ability to engage in complementary therapies. Moreover, combining neuromodulation with other treatments may result in beneficial synergistic effects at cognitive and neural levels. For example, combining neuromodulation and cognitive training has been shown to be beneficial for major depression and Alzheimer’s disease, when the training involves exercising a cognitive skill required for recovery. For patients with addictions, using neuromodulation techniques to induce plasticity whilst exercising key cognitive skill such as response inhibition, extinction learning, or cognitive re-framing, may have greater potential to modify neural networks and cognitive processes driving addictions, and potentially induce greater long-term effects (Rabey et al. 2013; Segrave et al. 2014).

## Future Perspective

A way forward in the application of neuromodulation for addiction might be to tailor these interventions to individual patients or subgroups of patients based on cognitive or neural profiles. First, this can be done by targeting specific cognitive candidates that are likely to map more closely to neural abnormalities than general addiction outcomes as craving or drug use. For instance, an overreliance on negative-reinforcement may indicate modulation of anti-reward brain network, whereas high impulsive patients may benefit more from targeting regions involved in cognitive control. Moreover, profiling based on neural functioning might be another way to investigate for which patients’ specific types of neuromodulation are most effective. Although few studies in the addiction field investigated or found effects on specific cognitive processes and none used profiling, studies in other fields show reason to be optimistic about this approach. A study that focused on rTMS for depression found that based on MRI resting state scans depressed patients can be divided in four subtypes related to specific symptom profiles that predict the response on rTMS treatment (Drysdale et al. 2016). Additionally, for neurofeedback approaches there are indications that certain participants are better able to modulate their brain activity. Better prediction of which patients will be successful can lead to better targeted neurofeedback treatment which can be especially important for the more expensive fMRI neurofeedback. A possible cheap method of predicting patients’ success is selecting patients that are able to modulate their temperature with biofeedback (Hartwell et al. 2013)

Moreover, there are encouraging developments in other psychiatric indications for neuromodulation that show how the interaction between neurobiological science and the application of neuromodulation can mutually advance each other. For instance, a German research group found a more optimal new target for the treatment of DBS in depression, the medial forebrain bundle, based on translational neurobiological research (Bewernick et al. 2017; Coenen et al. 2011). A different group found that scans informing about structural brain connectivity could help predict the optimal DBS target for individual patients with depression (Riva-Posse et al. 2018). These studies in other psychiatric disorders show a possible way forward to improve future application of neuromodulation in addiction treatment.
